# Qiangji Decoction Alleviates Neurodegenerative Changes and Hippocampal Neuron Apoptosis Induced by D-Galactose *via* Regulating AMPK/SIRT1/NF-κB Signaling Pathway

**DOI:** 10.3389/fphar.2021.735812

**Published:** 2021-09-23

**Authors:** Li-Ling He, Yun-Cui Wang, Ya-Ting Ai, Ling Wang, Si-Meng Gu, Ping Wang, Qing-Hua Long, Hui Hu

**Affiliations:** ^1^ School of Basic Medicine, Hubei University of Chinese Medicine, Wuhan, China; ^2^ School of Nursing, Hubei University of Chinese Medicine, Wuhan, China; ^3^ Department of Psychology, Jiangsu University Medical School, Zhenjiang, China

**Keywords:** Qiangji Decoction, brain aging, D-galactose, AMPK/SIRT1/NF-κB signaling pathway, neuroinflammation, neurodegenerative changes

## Abstract

Qiangji Decoction (QJD), a classic formula, has been widely used to treat brain aging–related neurodegenerative diseases. However, the mechanisms underlying QJD’s improvement in cognitive impairment of neurodegenerative diseases remain unclear. In this study, we employed D-galactose to establish the model of brain aging by long-term D-galactose subcutaneous injection. Next, we investigated QJD’s effect on cognitive function of the model of brain aging and the mechanisms that QJD suppressing neuroinflammation as well as improving neurodegenerative changes and hippocampal neuron apoptosis. The mice of brain aging were treated with three different dosages of QJD (12.48, 24.96, and 49.92 g/kg/d, respectively) for 4 weeks. Morris water maze was used to determine the learning and memory ability of the mice. HE staining and FJB staining were used to detect the neurodegenerative changes. Nissl staining and TUNEL staining were employed to detect the hippocampal neuron apoptosis. The contents of TNF-α, IL-1β, and IL-6 in the hippocampus were detected by using ELISA. Meanwhile, we employed immunofluorescence staining to examine the levels of GFAP and IBA1 in the hippocampus. Besides, the protein expression levels of Bcl-2, Bax, caspase-3, cleaved caspase-3, AMPKα, p-AMPKα-Thr172, SIRT1, IκBα, NF-κB p65, p-IκBα-Ser32, and p-NF-κB p65-Ser536 in the hippocampus of different groups were detected by Western blot (WB). Our findings showed that the QJD-treated groups, especially the M-QJD group, mitigated learning and memory impairments of the model of brain aging as well as the improvement of neurodegenerative changes and hippocampal neuron apoptosis. Moreover, the M-QJD markedly attenuated the neuroinflammation by regulating the AMPK/SIRT1/NF-κB signaling pathway. Taken together, QJD alleviated neurodegenerative changes and hippocampal neuron apoptosis in the model of brain aging via regulating the AMPK/SIRT1/NF-κB signaling pathway.

## Introduction

Brain aging is the main factor inducing aging-related neurodegenerative diseases (Fung et al., 2020). With the rapid escalation of the aging population all over the world, the prevalence of chronic neurodegenerative diseases including mild cognitive impairment (MCI) and Alzheimer’s disease (AD) is increasing rapidly. Although we have made encouraging progress in the research of MCI and AD, its high prevalence has resulted in a heavy burden on the economy and society of the world. To date, we have no effective therapies against MCI and AD, and current drugs cannot fundamentally reverse the pathological process of MCI and AD (Yiannopoulou and Papageorgiou, 2020; Trevisan et al., 2021). Thus, the delay of brain aging has been recognized as a key to prevent the onset of MCI and AD.

The causes resulting in brain aging are complex, of which neuroinflammation is considered as the crucial culprit. The increasing researches have confirmed that neuroinflammation can damage the structure and function of the brain and finally result in hippocampal-dependent learning and memory impairment (Lima Giacobbo et al., 2019; Yahfoufi et al., 2020; Salami et al., 2021). Therefore, the inhibition of neuroinflammation has been regarded as an effective therapeutic intervention to alleviate the progression of chronic neurodegenerative diseases.

D-galactose (D-gal), a type of reducing sugar, has been commonly found to present as the lactose in the milk of mammals (Shwe et al., 2018). In general, the low dose of D-gal can be metabolized into galactose-1-phosphate. But at higher concentrations, D-gal can be converted to aldose and hydrogen peroxide, causing the disposition of superoxide anion and oxygen-derived free radicals in the brain, and finally lead to brain damage (Sun et al., 2018). Mounting studies have confirmed that chronic D-gal administration leads to the cognitive impairment of rodents by the accumulated oxidative stress, mitochondrial deficits, and neuroinflammation (Chen et al., 2019; Jeong et al., 2021; Oskouei et al., 2021). For these reasons, chronic administration of D-gal is well-established in the experimental rodent models of aging-related cognitive impairment and investigating the anti-aging pharmacological studies. Chronic D-gal administration can not only induce oxidative stress but also lead to neuroinflammation. Previous researches have pointed out that neuroinflammation acts as a contributor in accelerating and deteriorating the pathological process of brain aging. Cumulative reports have showed that chronic D-gal administration can suppress the AMPK/SIRT1 signaling pathway, thereby activating the NF-κB signaling pathway (Wang et al., 2020a; Lin et al., 2020; Wang et al., 2021). When the NF-κB signaling pathway is activated, it can induce neuroinflammation (Wang et al., 2020b; El-Far et al., 2020). However, once the AMPK/SIRT1 signaling pathway is activated, NF-κB is also inhibited accordingly, and finally alleviating cognitive impairment and neurodegenerative changes induced by D-gal.

From the view of traditional Chinese medicine, kidney deficiency is considered as an important factor causing brain aging, and tonifying kidney is a critical treatment method for anti–brain aging. Qiangji Decoction (QJD) is a classic formula created by Chen Shiduo in Qing Dynasty and can nourish kidneys to delay the process of brain aging. QJD comprised four raw herbs, namely, wine-steamed roots of *Rehmannia glutinosa* Libosch. (family: Scrophulariaceae, Shudihuang in Chinese), air-dried mature seeds of *Ziziphus jujuba* Mill. var. *spinosa* (Bunge) Hu ex H. F. Chou (family: Rhamanaceae, Suanzaoren in Chinese), air-dried tuberous roots of *Ophiopogon japonicus* (L. f) Ker-Gawl. (family: Liliaceae, Maidong in Chinese), air-dried roots and rhizomes of *Polygala tenuifolia* Wild., or *Polygala sibirica* L. (family: Polygalaceae, Yuanzhi in Chinese). Since the Qing Dynasty, QJD has been demonstrated to have a positive therapeutic effect on chronic neurodegenerative diseases including MCI and AD. A large body of clinical practice has confirmed that QJD can alleviate the aging-related cognitive impairment. At the same time, animal experiments have also verified that QJD can alleviate the cognitive impairment and neurodegenerative changes induced by D-gal and scopolamine (Gu., et al., 2008; Li., et al., 2008). However, the molecular mechanism underlying QJD’s anti–brain aging is still unclear, so the present study was designed to evaluate whether QJD could mitigate the cognitive impairment induced by D-gal and then the molecular mechanisms that QJD inhibited neurodegenerative changes through anti-neuroinflammation were explored.

## Materials and Methods

### Animals

A total of 60 eight-week-old male C57BL/6 mice with specific pathogen free (SPF) were used in this study, weighing about 25 ± 2 g, which were obtained from Liaoning Changsheng Biotechnology Co., Ltd. (Benxi, Liaoning, China; Certification number: SCXK 2020-0001). The experimental animals used in this study were housed in the Center Laboratory of Chinese Medicine of Hubei University of Chinese Medicine. The standard laboratory was controlled at the temperature of 23 ± 2°C, 60% relative humidity, and 12:12 h lightdark cycle. During the experiment, the animals had free access to a standard diet and water. The approval of animal feeding and experiment protocol were obtained from the Animal Ethics Review Committee of Hubei University of Chinese Medicine (No. 8217150845) and conducted in accordance with the ethical guidelines.

### Drugs

Qiangji Decoction (QJD) comprised four raw herbs, including wine-steamed roots of *Rehmannia glutinosa* Libosch. (30 g), air-dried mature seeds of *Ziziphus jujuba* Mill. var. *spinosa* (Bunge) Hu ex H. F. Chou (30 g), air-dried tuberous roots of Ophiopogon japonicus (L. f) Ker-Gawl. (30 g), air-dried roots and rhizomes of *Polygala tenuifolia* Wild. or *Polygala sibirica* L. (6 g), which were provided by Jinpai Chizhengtang Pharmaceutical Co., Ltd. (Huangshi, Hubei, China). All raw herbs were authenticated by Prof. Qiu-yun You, a pharmacologist from Hubei University of Chinese Medicine, and identified in accordance with the Chinese Pharmacopoeia. Metformin hydrochloride extended-release tablets (0.5 g/tablet, batch number: ABU7707) were obtained from Sino-American Shanghai Squibb Pharmaceutical Co., Ltd. (Shanghai, China).

### Preparation of QJD Extract and Metformin

According to the methods described in our previous report with minor modifications, the stock solution of QJD was prepared (Long et al., 2021). In general, the raw herbs were crushed into pieces, and the mixtures of raw herbs were soaked in water (1:8, w/v) for 30 min. Subsequently, the mixed herbs were decocted for 30 min. After filtration, the residue was decocted for 20 min (1:6, w/v). The two filtrates were mixed and refluxed in the rotary evaporators (QYMD-60, Qi yu industry co., LTD., Shanghai, China) for 1.5 h, and finally concentrated into a stock solution. Metformin was dissolved in saline and prepared as a 0.01 g/ml stock solution.

### Reagents and Antibodies

D-galactose (D-gal), purity>99.0%, was supplied by Solarbio Science and Technology Co., Ltd. (Beijing, China; D8310). HE staining solution, Nissl staining solution, and Fluorescein (FITC) TUNEL Cell Apoptosis Detection Kit were obtained from Servicebio Technology Co., Ltd. (Wuhan, Hubei, China; G1005, G1036, and G1501, respectively). Fluoro-Jade B (FJB) staining kit was obtained from Merck-Millipore (Darmstadt, Germany; AG310). ELISA Kits for the detection of TNF-α, IL-1β, and IL-6 were supplied by ABclonal technology (Wuhan, Hubei, China; RK00027, RK00006, and RK00008, respectively). Rabbit monoclonal anti–Bcl-2, rabbit monoclonal anti-Bax, rabbit monoclonal anti–caspase-3, rabbit polyclonal anti–cleaved caspase-3, rabbit monoclonal anti–total-AMPKα, rabbit monoclonal anti–phospho-AMPKα (Thr172), rabbit monoclonal anti-SIRT1, rabbit monoclonal anti–*β*-actin, and HRP-linked goat anti-rabbit IgG were purchased from ABcam (Cambridge, MA, USA; ab32124, ab32503, ab32351,ab2302, ab32047, ab133448, ab110304, ab8227, and ab6721, respectively). Rabbit polyclonal anti-GFAP, rabbit monoclonal anti-IBA1, and Cy3-conjugated goat anti-rabbit IgG were purchased from ABclonal technology (Wuhan, Hubei, China; A0237, A1527, and AS007, respectively). Rabbit monoclonal anti–total-IκBα, rabbit monoclonal anti–phospho-IκBα (Ser32), rabbit monoclonal anti–total-NF-κB p65, and rabbit monoclonal anti–phospho-NF-κB p65 (Ser536) were purchased from Cell Signaling Technology (Beverly, MA, USA; #4812, #2859, #8242, and #3033, respectively).

### Experimental Model and QJD Administration

**FIGURE 1:**
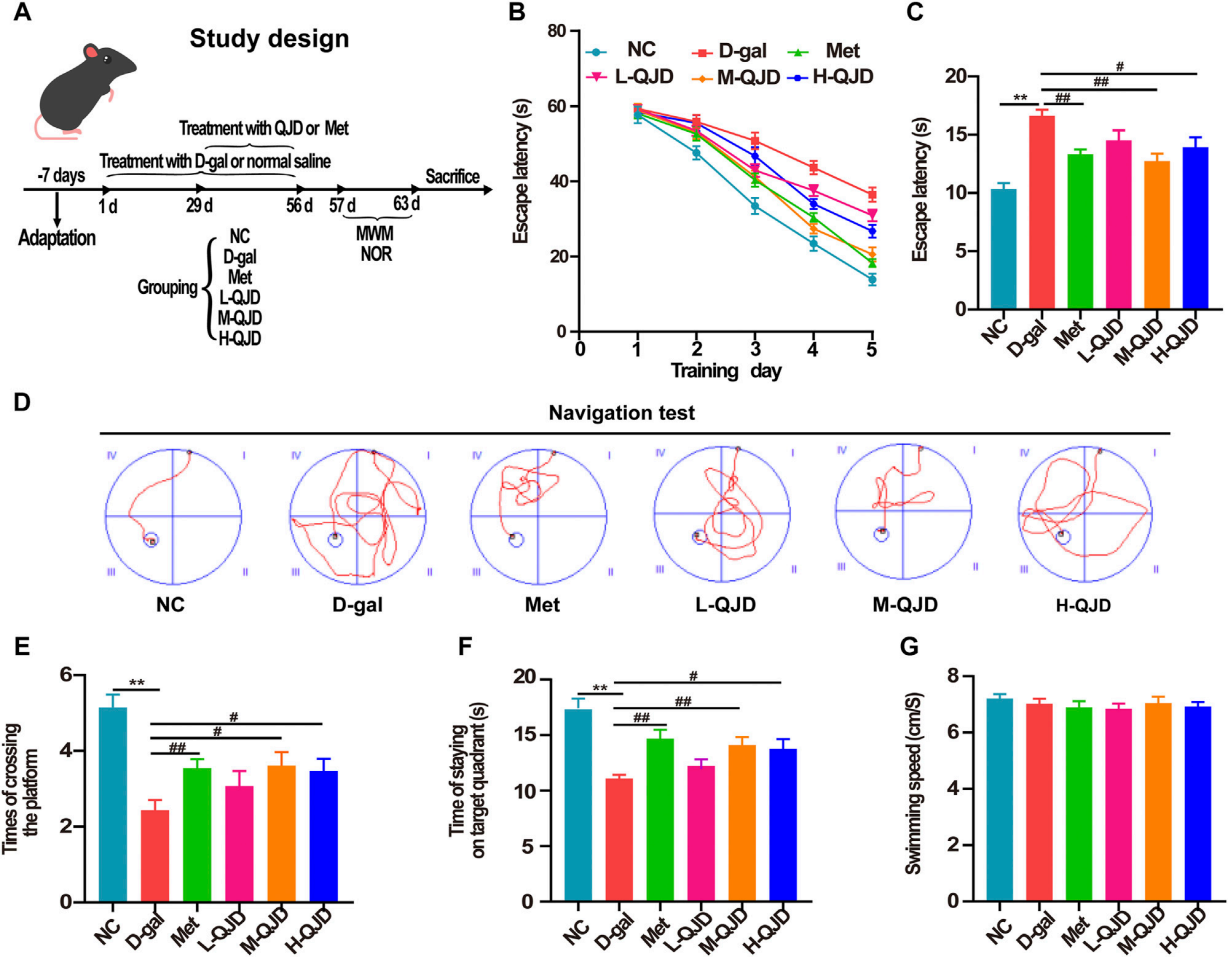
QJD alleviated learning and memory impairments in D-gal–induced mice. **(A)** Study design. **(B)** Mean escape latency to the platform of each group during the training session of five consecutive days. **(C)** Escape latency to the platform of each group in the navigation test. **(D)** Representative path tracings of each group in the navigation test. **(E)** The number of crossing platform in the permitted 60 s. **(F)** Time of staying on the target quadrant in the permitted 60 s. **(G)** The mean swimming speed of mice in all groups. All data were presented as mean ± SEM (*n* = 10/group). **p < 0.01 vs. NC group; ^#^ *p* < 0.05 and ^##^ *p* < 0.01 vs. D-gal group.

After the acclimation, the mice were randomly assigned to the following six different groups of 10 animals each, namely, negative control group (NC), D-gal group (D-gal), metformin group (Met), low dose of QJD group (L-QJD), middle dose of QJD group (M-QJD), and high dose of QJD group (H-QJD). With reference to the previous reports (Baeta-Corral et al., 2018; Ullah et al., 2020; Ahmad et al., 2021), the mice in the negative control group were administered with 0.9% saline by subcutaneous injection for 8 weeks, while the other groups were administered with D-gal (100 mg/kg/d). Based on the previous reports and clinical equivalent doses (Farr et al., 2019; Li et al., 2019), the therapeutic dose of the metformin group and QJD-treated group was confirmed accordingly. From the fifth to the eighth week, the NC group and the D-gal group received the same amount of normal saline by oral administration, the metformin group was given metformin (200 mg/kg/d), and the QJD-treated groups were treated with three different doses of QJD extract (L-QJD: 12.48 g/kg/d, M-QJD: 24.96 g/kg/d, H-QJD: 49.92 g/kg/d). As shown in Figure 1A, the study design of this research has been illustrated.

### Morris Water Maze

After the treatment was completed, the hippocampus-dependent learning and memory ability of mice in each group were evaluated by the Morris water maze (MWM) test. The equipment of the MWM test was given in our previous report, and it was performed with minor modifications (Long et al., 2021). Briefly, the MWM test contains two tests, namely, the navigation and spatial probe test. During the navigation test, every trained animal was given 60 s to search for the platform, and the escape latency was documented by the MWM software (version YH-MWM, Wuhan Yihong Technology Co., Ltd., China). If the experimental animals did not locate the platform within the allocated 60 s, they were manually led to the platform and allowed to remain on the platform for at least 15 s. After the completion of the navigation test, the hidden platform was retracted manually, and then the spatial probe test was executed. MWM software automatically recorded and analyzed the number of crossing the hidden platform in the spatial probe test. In addition, we also analyzed and counted the time of staying on the target quadrant and the mean swimming speed in each group.

### Hippocampal Tissue Collections

After the completion of MWM, four animals in each group were sacrificed, and then the brain tissues were collected to fix in 4% paraformaldehyde for HE staining, FJB staining, Nissl staining, TUNEL staining, and immunofluorescence staining. The remaining mice were anesthetized in the same way, and the hippocampal tissue was separated and split into two parts on a cold plate. One portion of the hippocampal tissue was used for ELISA, and another part was used for Western blot. After separation, the hippocampal tissue was in a cryopreservation tube and stored in liquid nitrogen for 30 min, finally kept at −80°C until further processing.

### HE Staining

The brain tissues were immersed in 4% paraformaldehyde at 4°C for 24 h and then processed into paraffin-embedded tissues. Subsequently, a microtome (CM 2016, Leica, Germany) was employed to cut the paraffin-embedded tissues into 5-μm-thick slices. The slices were dewaxed with xylene and dehydrated with gradient ethanol (100–75%). After being rinsed with double distilled water, the slices were placed in a solution of hematoxylin for 3–5 min and then stained with a solution of eosin for 5 min. Finally, neutral gum was used to mount the slices. The stained slices were photographed and analyzed with an upright optical microscope (ECLIPSE Ni-E, Nikon, Japan).

### Fluoro-Jade B Staining

Based on the manufacturer’s protocol and a previously described report, the FJB staining was carried out (Xie et al., 2019). Briefly, the prepared sections were immersed in xylene for 15 min. Subsequently, the slices were placed in a gradient ethanol of 85 and 75% for dehydration for 5 min each. The slices were rinsed in 0.1 M PBS for 1–2 min and then incubated to a solution of final working concentration of FJB for 10 min. The cell nuclei were labeled by 4 ′6-diamidino-2-phenylindole (DAPI). After FJB staining, the slices were visualized and photographed under an upright fluorescence microscope (DP72, Olympus, Japan), and then the number of FJB-positive cells were measured by using Image-Pro Plus 7.0 software (Media Cybernetics, Inc., Rockville, MD, USA).

### Nissl Staining

The paraffin-embedded slices were dewaxed with xylene for 5 min. Later, the dewaxed slices were dehydrated in a gradient ethanol series with decreasing concentration for 5 min (100–75%). After being dehydrated, the slices were treated with Nissl staining solution at 37°C for 30 min and then washed in 0.1 M PBS for 5 min. Subsequently, the paraffinized slices were incubated in 70% alcohol differentiation at 37°C for 15 s. The sections were dehydrated with gradient alcohol (70–95%) for 2 min and finally covered with neutral gum. The images were photographed and analyzed with an imaging system (BX50, Olympus, Japan). Subsequently, the number of neurons in the hippocampus was analyzed by Image-Pro Plus 7.0 software.

### TUNEL Staining

The TUNEL staining was employed to assess the level of hippocampal neuron apoptosis in each group, and it performed as described previously (Li et al., 2021). After being dewaxed and rehydrated, the paraffin-embedded slices were incubated with proteinase K at room temperature for 25 min. After incubation, the sections were washed in 0.1 M PBS for 5 min and then incubated in permeabilize working solution at room temperature for 20 min. The slices are slightly dried and then incubated with TUNEL mixture at room temperature for 2 h. Subsequently, the cell nuclei were counterstained by using 4′ 6-diamidino-2-phenylindole (DAPI), and the slices were finally mounted with anti-fade mounting medium. The slices were observed under an upright fluorescence microscope (DP72, Olympus, Japan). In this study, we used Image-Pro Plus 7.0 software to measure the number of TUNEL-positive cells, so as to assess whether QJD could improve the hippocampal neuronal apoptosis.

### Immunofluorescence Staining

Based on the previous report, the immunofluorescence staining was conducted with minor modifications (Rong et al., 2019). In brief, the slices were dewaxed with xylene and dehydrated with gradient ethanol (100–75%), and then washed in 0.1 M PBS for 5 min. Next, the dehydrated slices were placed in EDTA antigen retrieval buffer for 8 min at a sub-boiling temperature. Subsequently, the slices were blocked in 3% bovine serum albumin (BAS). After washing three times using 0.1 M PBS 3 min, the primary antibody (IBA1, 1:100; GFAP, 1:200) was selected to incubate the slices overnight at 4°C. The following day, the Cy3-conjugated secondary antibody (1:200) was added to incubate at 37°C for 50 min. DAPI was employed to label the cell nuclei, and the slices were finally mounted with anti-fade mounting medium. In this study, we used Image-Pro Plus 7.0 software to calculate the mean fluorescence intensity of images.

### Enzyme-Linked Immunosorbent Assay

To evaluate whether QJD could inhibit the neuroinflammation, we employed enzyme-linked immunosorbent assay (ELISA) kits to estimate the contents of pro-inflammatory factors in hippocampal tissue, such as TNF-α, IL-1β, and IL-6. In brief, the separated hippocampus was immersed in PBS (1:8, w/v), homogenized in a cold tissue homogenizer (JXFSTPRP-CL-24, Tuohe Electromechanical Technology Co., Ltd., Shanghai, China), followed by centrifugation (12,000 rpm) at 4°C for 10 min. The supernatant was then collected. Subsequently, the level of protein was quantified using the BCA Protein Assay kit. Finally, the contents of TNF-α, IL-1β, and IL-6 in hippocampus were detected using ELISA kits, following the manufacturer’s protocol.

### Western Blot

In brief, the separated hippocampus was lysed in RIPA lysis buffer (1:8, w/v) containing phenylmethylsulfonyl fluoride (PMSF) and phosphatase inhibitors to extract the total proteins of hippocampus. After homogenizing, the hippocampal homogenates were subjected to centrifugation (12,000 rpm) at 4°C for 30 min, and the supernatants were collected. Subsequently, BCA Protein Assay kit was used to calculate the protein concentration of each sample. After that, the 10–12% separation gel buffer and 5% stacking gel buffer were prepared, and the protein of each sample (30 μg) was separated with SDS-PAGE. The protein was then transferred to the PVDF membranes (Millipore, Darmstadt, Germany). After blocked in 5% nonfat milk at room temperature for 30 min, the membranes were incubated with primary antibodies including anti–Bcl-2 (1:1,000), anti-Bax (1:1,000), anti–cleaved caspase-3 (1:1,000), anti-AMPKα (1:1,000), anti–*p*-AMPKα-Thr172 (1:1,000), anti-SIRT1 (1:1,000), anti-IκBα (1:1,000), anti–*p*-IκBα-Ser32 (1:1,000), anti–NF-κB p65 (1:1,000), anti–p-NF-κB p65-Ser536 (1:1,000), and anti–β-actin (1:1,000) at 4°C overnight. The next day, the membranes were incubated with HRP-linked secondary antibody (1:10,000) at room temperature for 30 min. After washing three times with TBST, ECL kit was used to capture the blots, and the band intensity of each sample was quantified by using the Image J software (Bethesda, United States).

### Statistical Analysis

All experimental data were dealt by using Statistical Analysis System software (SAS, version 9.4, SAS Institute Inc., Cary, NC, USA), and the results were expressed as mean ± standard error of the mean (SEM). The two-way analysis of variance (ANOVA) with repeated measures was used to analyze the escape latency, and the remaining data were analyzed by one-way ANOVA. If the variance was homogeneous, the Bonferroni post hoc test was adopted. If not, the Tamhane T2 test was used. When the *p*-value was less than 0.05, values were considered statistically significant.

## Results

### QJD Alleviates Learning and Memory Impairments in D-Gal–Induced Mice

MWM is one of the most widely used behavioral testing methods, which has been commonly used to evaluate the learning and memory ability of brain aging–related neurodegenerative diseases (Garthe and Kempermann, 2013; Wahl et al., 2017). Accordingly, we assessed the mice’s spatial learning ability in each group by using the navigation test. During the navigation test, there was no obvious alteration in the escape latency among all groups during 3 days of consecutive training (*p* > 0.05, Figure 1B). Starting from the fourth day, the D-gal treatment significantly increased the escape latency, whereas this phenomenon was significantly reversed by the metformin, M-QJD, and H-QJD (*p* < 0.05 or 0.01; Figures 1B, C). The representative path tracings of each group in the navigation test were presented in Figure 1D, which indicated the M-QJD significantly shortened the escape latency in D-gal-induced mice. Besides, we examined the spatial memory ability of mice by using the spatial probe test. According to the spatial probe test, we found the M-QJD group and H-QJD group had a higher number of crossing platform when compared to the D-gal group (*p* < 0.05 or 0.01; Figure 1E). Meanwhile, the M-QJD group and H-QJD group significantly increased the time that mice spent in the target quadrant (*p* < 0.05 or 0.01; Figure 1F). Nevertheless, the L-QJD group did not significantly change the abovementioned results (*p* > 0.05; Figures 1E,F). In mean swimming speed, no statistical difference was found among all groups (*p* > 0.05; Figure 1G), which implies that mice’s swimming ability had no difference. These results indicated that chronic D-gal administration could induce the cognitive impairment, and the QJD, especially the M-QJD, reversed this phenomenon, whereas the L-QJD did not mitigate the cognitive impairment in D-gal–induced mice.

### QJD Alleviates the Pathological Alterations in the Hippocampus of D-Gal–Induced Mice

**FIGURE 2:**
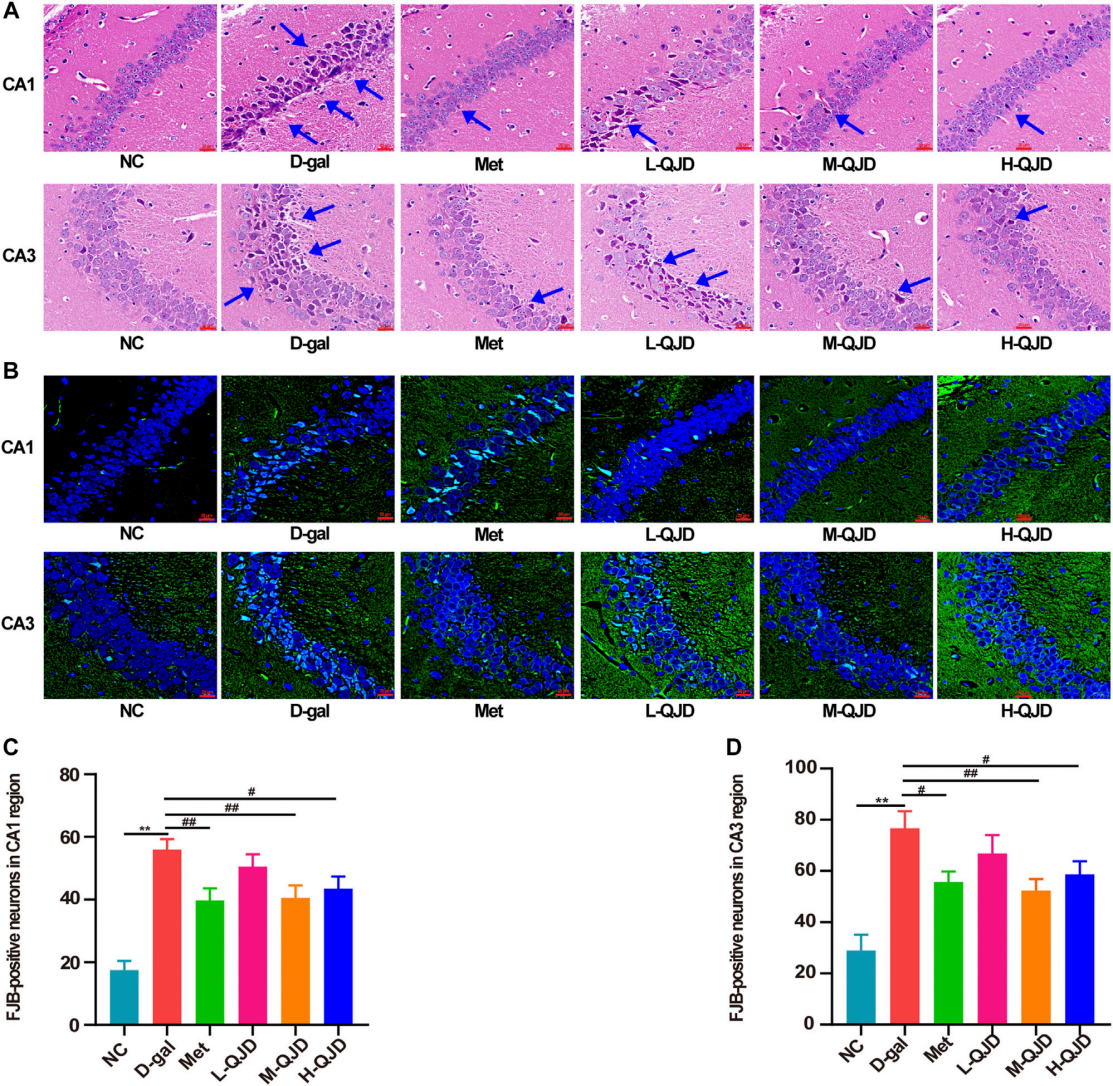
QJD alleviated the pathological alterations in the hippocampus of D-gal–induced mice. **(A)** Representative HE staining images in the hippocampus of CA1 and CA3 regions (magnification × 400; scale bar = 20 μm). **(B)** Representative FJB staining images (labeled in green) in the hippocampus of CA1 and CA3 regions (magnification × 400; scale bar: 20 μm). **(C)** Number of FJB-positive neurons in the hippocampus CA1 region. **(D)** Number of FJB-positive neurons in the hippocampus CA3 region. The blue arrows indicate damaged neurons in the hippocampus. All data were presented as mean ± SEM (*n* = 4/group). **p < 0.01 vs. NC group; ^#^ *p* < 0.05 and ^##^ *p* < 0.01 vs. D-gal group.

The MWM test results show that the QJD could alleviate the cognitive deficits induced by D-gal, and thereby, the HE staining and FJB staining were utilized to evaluate whether QJD could rescue the pathological alterations of the hippocampus induced by D-gal. In HE staining, we noticed that the D-gal group’s neurons in the hippocampal CA1 and CA3 regions were degenerated with dark staining, deformed, and denatured nuclei in comparison with the NC group (Figure 2A). However, the neuronal damages in these regions of the M-QJD group and H-QJD group were decreased in comparison to the D-gal group, and the stained neurons and deformed nuclei were also alleviated. In addition, neurodegenerative changes in the hippocampus were detected by using FJB staining. As showed in FJB staining, compared with the NC group, there were remarkably increased FJB-positive cells in the hippocampal CA1 and CA3 regions of D-gal group (*p* < 0.01, Figures 2B–D). However, after treatment with M-QJD and H-QJD, the number of FJB-positive cells in these regions were significantly diminished (*p* < 0.05 or 0.01; Figures 2B–D). The above findings manifested that QJD could relieve the pathological alterations of hippocampus induced by D-gal.

### QJD Alleviates the Hippocampal Neuron Loss and Apoptosis in D-Gal–Induced Mice

**FIGURE 3:**
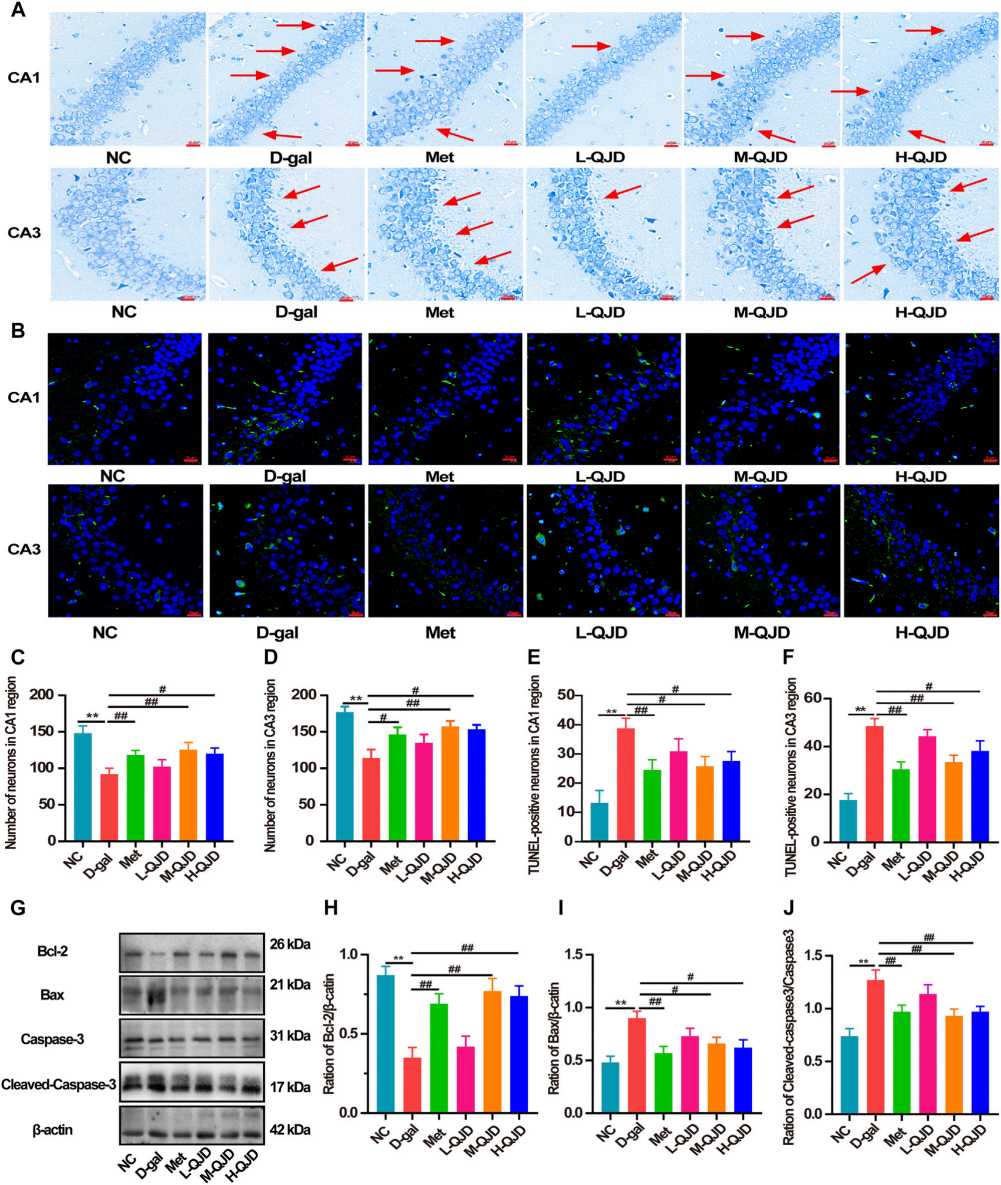
QJD alleviated the hippocampal neuron loss and apoptosis in D-gal–induced mice. **(A)** Representative Nissl staining images in the hippocampus of CA1 and CA3 regions (magnification × 400; scale bar = 20 μm). **(B)** Representative TUNEL staining images (labeled in green) in the hippocampus of CA1 and CA3 regions (magnification × 400; scale bar = 20 μm). **(C)** Number of neurons in the hippocampus CA1 region. **(D)** Number of neurons in the hippocampus CA3 region. **(E)** Number of TUNEL-positive neurons in the hippocampus CA1 region. **(F)** Number of TUNEL-positive neurons in the hippocampus CA3 region. **(G)** Representative Western blot bands showing the protein expression levels of Bcl-2, Bax, caspase-3 and cleaved caspase-3 in the hippocampus. **(H)** Relative protein expression level of Bcl-2. **(I)** Relative protein expression level of Bax. **(J)** Relative protein expression level of cleaved caspase-3/caspase-3. The red arrows indicate the hippocampal neuron loss. All data were presented as mean ± SEM (*n* = 4 or 6/group). **p < 0.01 vs. NC group; ^#^ *p* < 0.05 and ^##^ *p* < 0.01 vs. D-gal group.

We also employed Nissl staining and TUNEL staining to investigate whether QJD could improve the hippocampal neuron loss and apoptosis in D-gal–induced mice. In Nissl staining, we found that there was a decreased number of neurons in the CA1 and CA3 regions, when compared to the NC group (*p* < 0.01; Figures 3A,C,D). Whereas compared to the D-gal group, the decreased neurons were rescued by the M-QJD and the H-QJD (*p* < 0.05 or 0.01; Figures 3A,C,D). We also used the TUNEL staining to evaluate whether QJD could restrain neuronal apoptosis and quantified the number of TUNEL-positive cells by using Image-Pro Plus 7.0 software. The TUNEL staining results showed that the decreased TUNEL-positive neurons in the CA1 and CA3 regions were inhibited by the M-QJD and the H-QJD (*p* < 0.05 or 0.01; Figures 3B,E,F). In addition, the apoptotic markers including Bcl-2 (anti-apoptotic protein), Bax (apoptosis regulator), and cleaved caspase-3 (pro-apoptotic protein) were detected by Western blot (Ullah et al., 2020). Our finding verified that the D-gal treatment could upregulate the protein levels of Bax and cleaved caspase-3 in the hippocampus compared to the NC group (*p* < 0.01; Figures 3G,I,J), while the protein levels of Bcl-2 were significantly downregulated (*p* < 0.01; Figures 3G,H). After 4 weeks of M-QJD and H-QJD treatment, the above phenomenon was reversed (*p* < 0.05 or 0.01; Figures 3G–J). These results implied that the QJD could alleviate the hippocampal neuron loss and apoptosis in D-gal–induced mice.

### QJD Alleviates the Neuroinflammation Through Suppressing Microglial and Astrocytes Activation in D-Gal–Induced Mice

**FIGURE 4:**
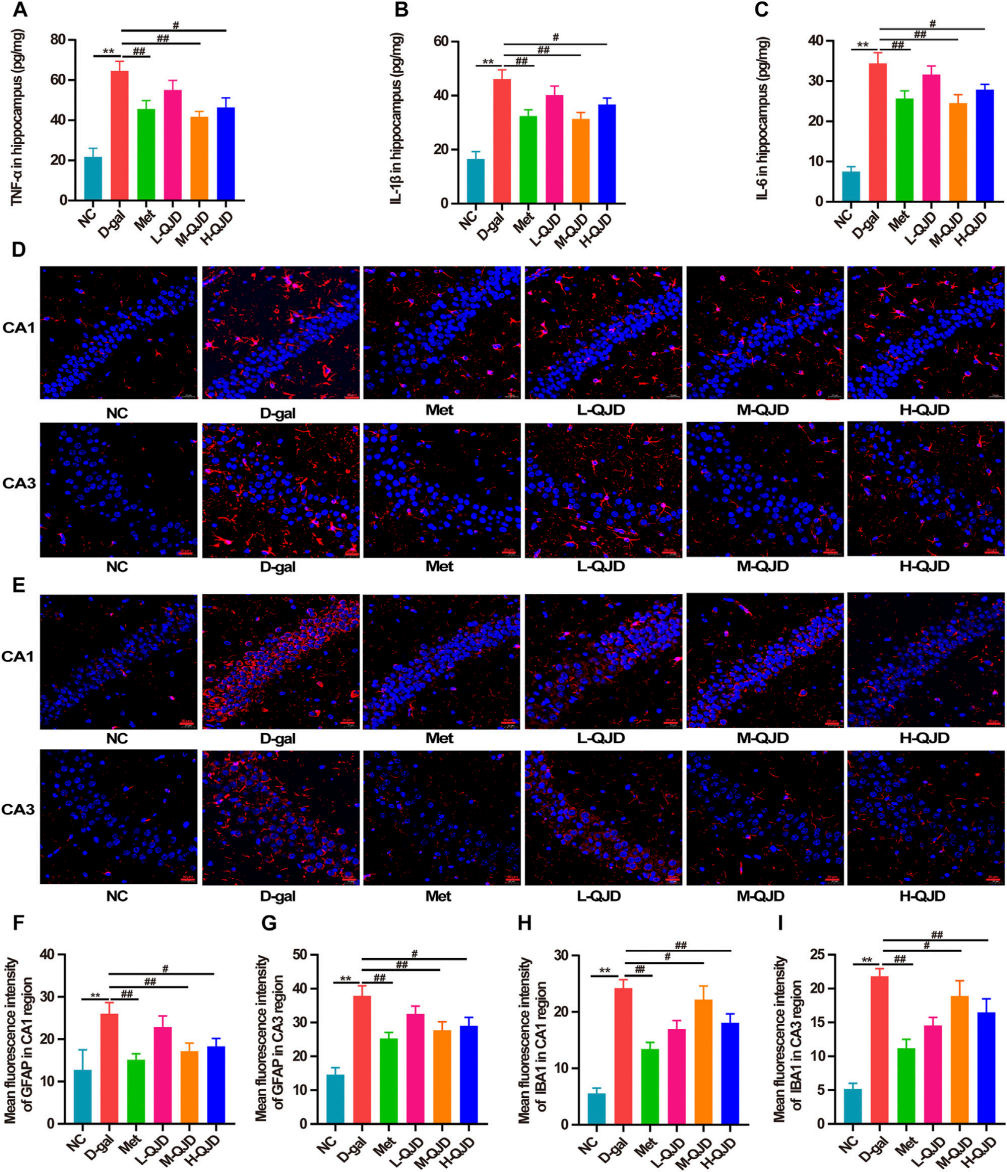
QJD alleviated the neuroinflammation through suppressing microglial and astrocytic activation in D-gal–induced mice. **(A)** Protein expression of TNF-α in hippocampus. **(B)** Protein expression of IL-1β in hippocampus. **(C)** Protein expression of IL-6 in hippocampus. **(D)** Representative immunofluorescence staining images of GFAP (labeled in red) in the hippocampus of CA1 and CA3 regions (magnification × 400; scale bar = 20 μm). **(E)** Representative immunofluorescence staining images of IBA1 (labeled in red) in the hippocampus of CA1 and CA3 regions (magnification × 400; scale bar = 20 μm). **(F)** Mean fluorescence intensity of GFAP in the hippocampus of CA1 region. **(G)** Mean fluorescence intensity of GFAP in the hippocampus of CA3 region. **(H)** Mean fluorescence intensity of IBA1 in the hippocampus of CA1 region. **(I)** Mean fluorescence intensity of IBA1 in the hippocampus of CA3 region. All data were presented as mean ± SEM (*n* = 4 or 6/group). **p < 0.01 vs. NC group; ^#^ *p* < 0.05 and ^##^ *p* < 0.01 vs. D-gal group.

Neuroinflammation acts as the contributor in accelerating the pathological progression of aging-related neurodegenerative diseases, and the increasing researches have demonstrated that chronic D-gal administration can stimulate the microglia and astrocytes activation in central nervous system, thereby inducing chronic neuroinflammation (Lu et al., 2010; Cao et al., 2019; Yahfoufi et al., 2020). Therefore, in this study, we measured the contents of TNF-α, IL-1β, and IL-6 in the hippocampus through ELISA. When compared to the D-gal group, the NC group had a lower level of TNF-α, IL-1β, and IL-6 (*p* < 0.01; Figures 4A–C). However, after treatment with M-QJD and H-QJD, these pro-inflammatory cytokines were suppressed (*p* < 0.05 or 0.01; Figures 4A–C). The microglia and astrocytes activation is the critical factor for the release of pro-inflammatory cytokines. Growing researches have confirmed that chronic D-gal administration can induce the microglia and astrocytes activation, thereby accelerating the release of pro-inflammatory cytokines (Lu et al., 2010; Jeong et al., 2021; Oskouei et al., 2021). So, we used the immunofluorescence staining to detect the levels of IBA1 and GFAP. Our findings showed that the D-gal group had higher fluorescence intensity of IBA1-positive microglia and GFAP-positive astrocytes (*p* < 0.01; Figures 4D–I), when compared to the NC group. After treatment with M-QJD and H-QJD, the fluorescence intensity of IBA1-positive microglia and GFAP-positive astrocytes was significantly decreased (*p* < 0.05 or 0.01; Figures 4D–I). Collectively, these results manifested that QJD could alleviate chronic neuroinflammation through suppressing the activation of microglial and astrocytes in D-gal–induced mice.

### QJD Activates the AMPK/SIRT1 Signaling Pathway in the Hippocampus of D-Gal–Induced Mice

**FIGURE 5:**
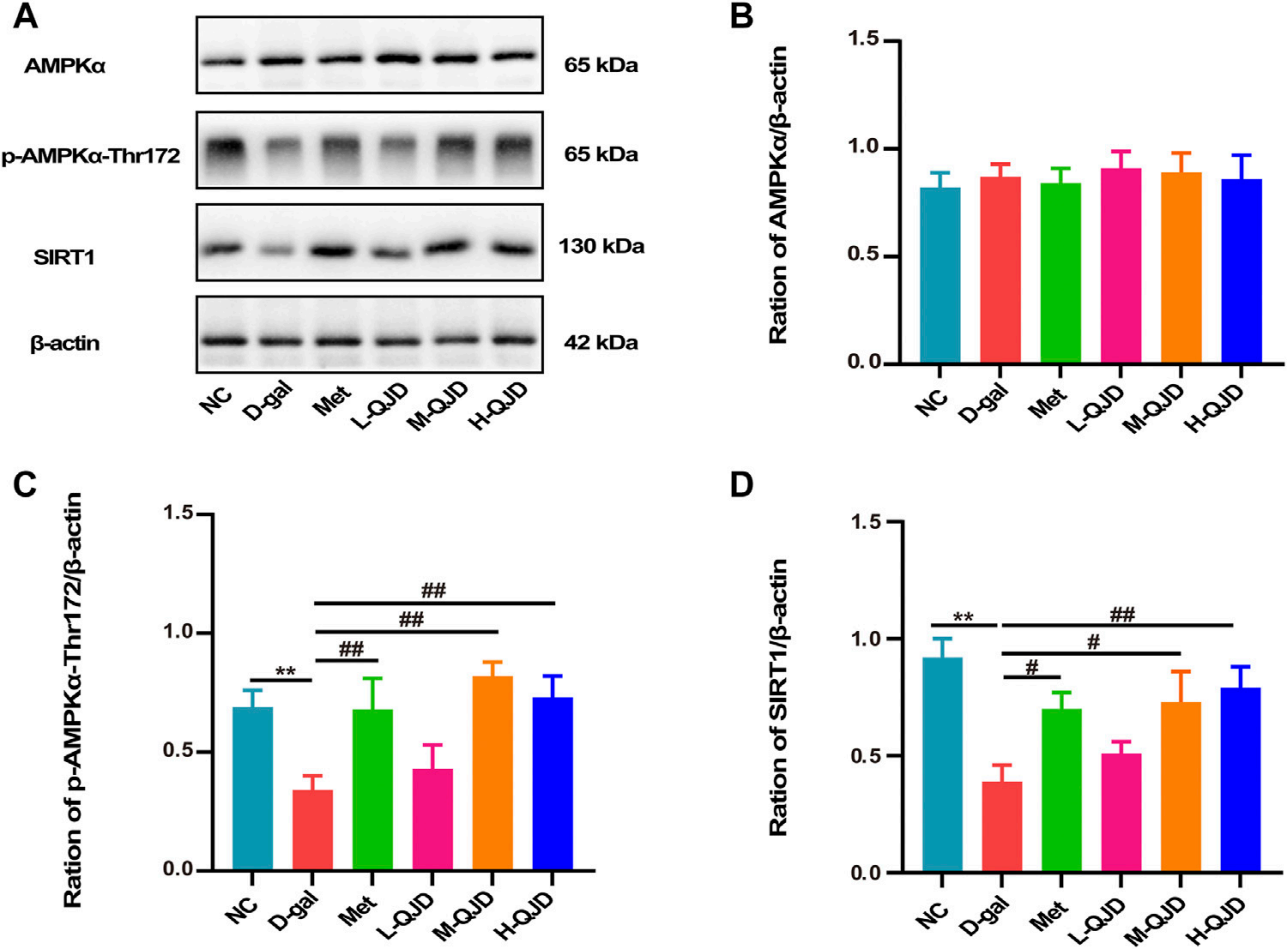
QJD activated the AMPK/SIRT1 signaling pathway in the hippocampus of D-gal–induced mice. **(A)** Representative Western blot bands showing the protein expression levels of AMPKα, *p*-AMPKα-Thr172, and SIRT1 in the hippocampus. **(B)** Relative protein expression level of AMPKα. **(C)** Relative protein expression level of *p*-AMPKα-Thr172. **(D)** Relative protein expression level of SIRT1. All data were presented as mean ± SEM (*n* = 6/group). **p < 0.01 vs. NC group; ^#^ *p* < 0.05 and ^##^ *p* < 0.01 vs. D-gal group.

In the aging-related neurodegenerative diseases, the AMPK/SIRT1 signaling pathway acts as a crucial role in regulating energy metabolism, apoptosis as well as neuroinflammation (Domise and Vingtdeux, 2016; Chen et al., 2020). Previous research has confirmed that the inactivation of this signal pathway can accelerate neuroinflammation, while the activation of this signal pathway can naturally inhibit neuroinflammation (Peixoto et al., 2017). In this study, we examined the levels of AMPK and SIRT1 and the phosphorylation of APMK in the hippocampus by using Western blot. Compared with NC group, the levels of SIRT1 and the phosphorylation of APMK in the D-gal group were significantly decreased (*p* < 0.01; Figures 5A–D), which indicated that the AMPK/SIRT1 signaling pathway was inhibited by D-gal. After treatment with QJD, the M-QJD and H-QJD had a higher levels of SIRT1 and the phosphorylation of APMK, when compared to the D-gal group (*p* < 0.05 or 0.01; Figures 5A–D). Furthermore, we noticed that the H-QJD had the similar effect with M-QJD. Those results showed that QJD could activate the AMPK/SIRT1 signaling pathway in the hippocampus of D-gal–induced mice.

### QJD Inhibits the NF-κB Signaling Pathway in the Hippocampus of D-Gal–Induced Mice

**FIGURE 6:**
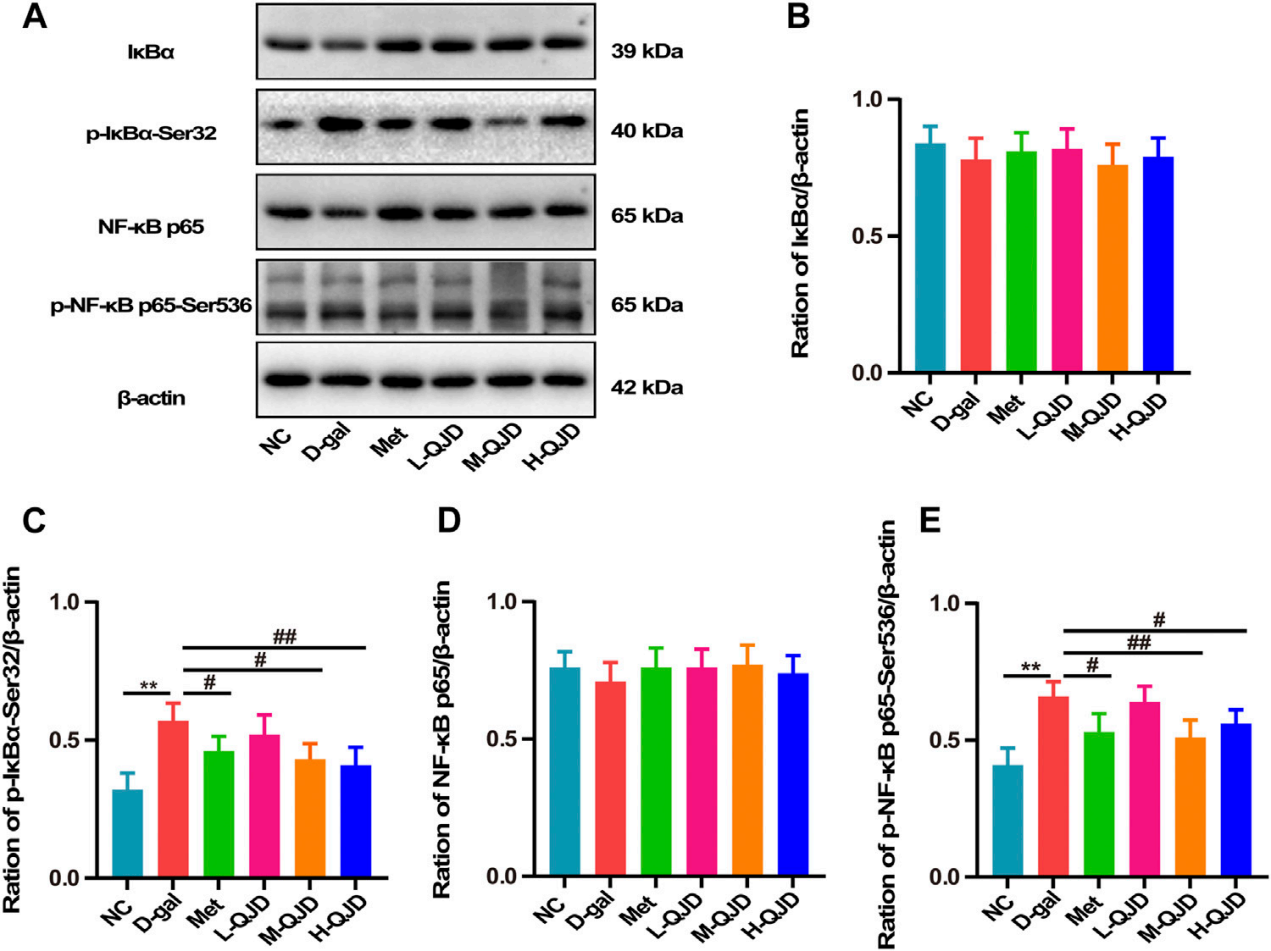
QJD inhibited the NF-κB signaling pathway in the hippocampus of D-gal–induced mice. **(A)** Representative Western blot bands showing the protein expression levels of IκBα, p-IκBα-Ser32, NF-κB p65, and p-NF-κB p65-Ser536 in the hippocampus. **(B)** Relative protein expression level of IκBα. **(C)** Relative protein expression level of *p*-IκBα-Ser32. **(D)** Relative protein expression level of NF-κB p65. **(E)** Relative protein expression level of p-NF-κB p65-Ser536. All data were presented as mean ± SEM (*n* = 6/group). **p < 0.01 vs. NC group; ^#^ *p* < 0.05 and ^##^ *p* < 0.01 vs. D-gal group.

AMPK/SITR1 signaling pathway is an important regulator of NF-κB, and the activity of NF-κB can be restrained by the AMPK/SITR1 signaling pathway. Growing researches have suggested that the activation of the AMPK/SITR1 signaling pathway can inhibit the activation of NF-κB, so we further investigate whether QJD alleviates the neuroinflammation through regulating the AMPK/SITR1–mediated NF-κB signaling pathway. In this study, we first used Western blot to examine the IκBα and NF-κB p65 in the hippocampus. Subsequently, the phosphorylated IκBα (Ser32) and NF-κB p65 (Ser536) were also detected by Western blot. As revealed by Figures 6A–E, the D-gal treatment significantly increased the levels of phosphorylated IκBα and phosphorylated NF-κB p65 in the hippocampus of the D-gal group, when compared to the NC group (*p* < 0.01). However, the H-QJD, especially the M-QJD, suppressed the levels of phosphorylated IκBα and phosphorylated NF-κB p65 (*p* < 0.05 or 0.01; Figures 6A–E). These data indicated that QJD could inhibit the NF-κB signaling pathway activation in the hippocampus of D-gal–induced mice.

## Discussion

Cognitive and memory deficits are one of the major clinical symptoms in brain aging. Numerous studies have confirmed that long-term D-gal subcutaneous injection (100 mg/kg) can induce memory impairment of rodents, so we employed the D-gal to establish the model of brain aging, and the mice were treated with D-gal by subcutaneous injection for 8 weeks in the present research (Chen et al., 2019; Jeong et al., 2021; Oskouei et al., 2021). Metformin is the activator of AMP-activated protein kinase (AMPK) (Steinberg and Carling., 2019). Simultaneously, previous studies have confirmed that metformin can alleviate the memory impairment of brain aging models in rodents by inhibiting oxidative stress and neuroinflammation, so it was used as a controlled drug in this research (Pan et al., 2016; Mudgal et al., 2019; Saffari et al., 2020). After treatment with metformin and QJD, we applied the MWM test to examine the learning and memory of mice in each group. As showed in Figure 1, there was no obvious difference in average escape latency to platform among all groups during the first to third days of the navigation test, whereas the D-gal group significantly increased the escape latency to the platform from the fourth day compared with the NC group. Taken together, our data implied that we have successfully established the model of brain aging by subcutaneous injection of D-gal. After 4-week treatment, the M-QJD group and the H-QJD group had shortened escape latency to platform compared with the D-gal group, while we did not find a considerable alteration in the L-QJD group. The navigation test showed that M-QJD and H-QJD could mitigate the spatial learning impairment of the model of brain aging. As for the spatial probe test, the H-QJD, especially the M-QJD, had significantly increased the number of crossing platform and time spent in the target quadrant, when compared with the D-gal group. Both of above data indicated that M-QJD and H-QJD could alleviate the spatial memory impairment in the model of brain aging. In addition, the metformin group has a similar effect to the M-QJD group. Collectively, the metformin and QJD, especially the M-QJD, could alleviate the cognitive impairment in the model of brain aging induced by D-gal.

The structure and function of neurons are critical for the hippocampus-dependent learning and memory, and the damage of neuronal structure and function can induce the learning and memory dysfunction. Growing researches have demonstrated that pathological alterations in hippocampus can be induced by long-term D-gal subcutaneous injection (Bei et al., 2018; Wang L. et al., 2020). To evaluate whether QJD can alleviate the pathological changes in the hippocampus induced by D-gal, we used HE staining to detect the pathologically degenerated neurons in the hippocampus. In the HE staining, we found that the D-gal group had obvious histopathological degenerative changes in hippocampal CA1 and CA3 regions. Furthermore, FJB staining was also employed to detect degenerative neurons, and the number of FJB-positive neurons in the hippocampus was analyzed by Image-Pro Plus 7.0 software. As showed in FJB staining, the D-gal group had a higher number of FJB-positive neurons in hippocampal CA1 and CA3 regions than the NC group. Therefore, the FJB staining’s results were consistent with the HE staining, both of which indicated that D-gal could induce hippocampal neurodegenerative changes. After treatment with QJD for 4 weeks, we find that the hippocampal neurodegenerative changes were improved compared to the D-gal group. The FJB staining also confirmed that QJD could reduce the number of FJB-positive cells of CA1 and CA3 regions in hippocampus. Taken together, the above experiments clearly suggested that QJD could alleviate the pathological alterations in the hippocampus induced by the D-gal.

Neuronal loss and apoptosis are other pathological features of the brain aging (Shruster et al., 2010; Grimm and Eckert, 2017). Thus, the Nissl staining and TUNEL staining were carried out to evaluate whether QJD could ameliorate the neuron loss and apoptosis, respectively. As showed in Nissl staining, compared with the NC group, the number of neurons in the hippocampal CA1 and CA3 regions in the D-gal group was decreased. However, we found that the number of TUNEL-positive cells in hippocampal CA1 and CA3 regions of the D-gal group was prominently higher than the NC group. In short, both Nissl staining and TUNEL staining demonstrated that long-term D-gal subcutaneous injection (100 mg/kg) can induce the neuron loss and apoptosis. After 4-week treatment, the H-QJD group, especially the M-QJD group, increased the neurons in the hippocampal CA1 and CA3 regions, whereas the TUNEL-positive cells in hippocampal CA1 and CA3 regions were significantly reduced. Thus, our present study indicated that QJD could inhibit neurodegenerative changes and hippocampal neuron apoptosis induced by D-gal. Besides, Western blot was carried out to detect the apoptotic markers including Bcl-2 (anti-apoptotic protein), Bax (apoptosis regulator), and cleaved caspase-3 (pro-apoptotic protein). We noticed that the QJD groups had lower protein expression level of Bax in hippocampus in comparison with the D-gal group, and protein expression level of cleaved caspase-3 in hippocampus showed a similar trend to the Bax. However, the M-QJD and H-QJD increased the protein expression level of Bcl-2. Collectively, the above data suggested that QJD could relieve the hippocampal neuron loss and apoptosis induced by the D-gal.

Neuroinflammation is significantly involved in aggravating cognitive impairment and neurodegenerative changes of brain aging (Singhal et al., 2014). Accumulated researches have confirmed that long-term D-gal subcutaneous injection can induce neuroinflammation by stimulating the activation of microglia and astrocytes (Lu et al., 2010; Jeong et al., 2021; Oskouei et al., 2021). Meanwhile, the activation of microglia and astrocytes will further trigger neuroinflammation, and finally releasing the pro-inflammatory factors, such as TNF-α, IL-1β, and IL-6, in D-gal–induced mice. Thus, we used ELISA to detect the levels of TNF-α, IL-1β, and IL-6 in the hippocampus. The ELISA results suggested that QJD-treated groups, especially the M-QJD remarkably, suppressed the contents of TNF-α, IL-1β, and IL-6 in the hippocampus, when compared with the D-gal group. Besides, the IBA1 and GFAP expression in the hippocampus of each group was examined through immunofluorescence staining. By analyzing the fluorescence intensity of microglia and astrocytes, we noticed that the M-QJD group and the H-QJD group had a decreased level of IBA1 and GFAP in the hippocampal CA1 and CA3 regions in comparison with the D-gal group. These obtained findings demonstrated that QJD could alleviate the neuroinflammation through suppressing microglial and astrocytes activation in D-gal–induced mice.

AMP-activated protein kinase (AMPK) known as the crucial factor in the regulation of cellular energy metabolism in hippocampal tissues, but it has been demonstrated to ameliorate brain aging by regulating energy metabolism, oxidative stress, neuroinflammation, apoptosis, and autophagy (Shah et al., 2017; Stern and Mcnew, 2021). AMPKα, the representative member of the AMPK family, and the phosphorylated AMPKα at the Thr172 site can inhibit neuroinflammation and apoptosis by activating silent information regulator of transcription 1 (SIRT1). In this research, we employed the Western blot to examine the protein expression level of *p*-AMPKα-Thr172 as well as a total of AMPKα and SIRT1 in the hippocampus of mice. Our findings showed that the D-gal group had a lower protein expression level of *p*-AMPKα-Thr172 and SIRT1 in comparison with the NC group. Thus, the present research suggested that the D-gal could suppress the AMPK/SIRT1 signaling pathway. After treatment with the QJD, compared with the D-gal group, we noticed that the protein expression level of *p*-AMPKα-Thr172 and SIRT1 was remarkably increased in the M-QJD group. Collectively, our study showed that QJD could activate the AMPK/SIRT1 signaling pathway.

Nuclear factor-kappa beta (NF-κB) can be regulated by the AMPK/SIRT1 signaling pathway, and the activation of the AMPK/SIRT1 signaling pathway can inhibit the NF-κB, thereby suppressing the neuroinflammation (Li et al., 2020; Zhang et al., 2020). Under physiological conditions, NF-κB combines with IκBα in the cytoplasm. However, IκBα can be phosphorylated at serine 32 site by TNF-α, IL-1β, and IL-6. Subsequently, phosphorylated IκBα further promotes the activation of NF-κB p65 at serine 536, thereby inducing the neuroinflammation (Sivandzade et al., 2019; Ding et al., 2020). Hence, we employed Western blot to detect the protein expression levels of *p*-IκBα-Ser32 and p-NF-κB p65-Ser536 as well as the total of IκBα and NF-κB p65. As shown in Figure 6, the increased *p*-IκBα-Ser32 and p-NF-κB p65-Ser536 were rescued in the M-QJD group and the H-QJD group. The above results showed that QID could mitigate neuroinflammation through inhibiting the NF-κB signaling pathway in D-gal–induced mice.

## Conclusions

In short, the present study showed that QJD alleviated cognitive impairment and neurodegenerative changes induced by D-gal. Furthermore, QJD attenuated the neuroinflammation by regulating the AMPK/SIRT1/NF-κB signaling pathway. Hence, our present study suggested that QJD may offer a promising therapeutic intervention in preventing cognitive impairment of brain aging.

## Data Availability

The original contributions presented in the study are included in the article/supplementary material, and further inquiries can be directed to the corresponding author/s.

## Abbreviation

AMPK, mammalian 5′-AMP-activated protein kinase; Bax, BCL-2–associated X protein; Bcl-2, B-cell lymphoma 2; DAPI, 4′ 6-diamidino-2-phenylindole; D-gal, D-galactose; FJB, Fluoro-Jade B; GFAP, glial fibrillary acid protein; HE, hematoxylin-eosin; IBAl, ionized calcium-binding adaptor molecule 1; IL-6, interleukin-6; IL-lβ, interleukin-l beta; IκBα, nuclear factor of kappa light polypeptide gene enhancer in B-cell inhibitor; MWM, Morris water maze; NF-κB, nuclear factor-kappa beta; PBS, phosphate-buffered saline; PVDF, polyvinylidene difluoride; QJD, Qiangji Decoction; SDS-PAGE, sodium dodecyl sulfate-polyacrylamide gel electrophoresis; SIRT1, silent information regulator of transcription 1; TNF-α, tumor necrosis factor-alpha; TUNEL, terminal deoxynucleotidyl transferase-mediated dUTP-biotin nick end labeling; WB, Western blot.
